# Effect of chronic lung diseases on angina pectoris among Indian adults: longitudinal ageing study in India

**DOI:** 10.1038/s41598-024-52786-x

**Published:** 2024-01-29

**Authors:** Waquar Ahmed, Priyanka Dixit

**Affiliations:** School of Health Systems Studies, Tata Institute of Social Sciences, Mumbai, India

**Keywords:** Geriatrics, Epidemiology, Respiratory tract diseases

## Abstract

The study aimed to evaluate the effect of chronic lung diseases, namely chronic obstructive pulmonary diseases (COPD) and asthma, on angina pectoris in individuals aged 45 years and above. Identifying vulnerable subpopulations suffering from COPD and asthma at higher risk of future cardiovascular events using the rose angina questionnaire is imperative for tailored primary and secondary prevention approaches. The present study utilizes the data from the Longitudinal Ageing Study in India, wave 1, conducted during 2017–2018. The sample size included 58,830 individuals aged 45 years and above. Angina was measured based on seven questions from Rose's questionnaires. Descriptive statistics and bivariate analysis were employed to examine the prevalence of angina among individuals with COPD and asthma. Further, multivariable logistic regression and propensity score matching (PSM) methods were used to assess the independent effect of COPD and asthma on angina after controlling the selected background characteristics. We employed PSM in two different models and included various additional factors in model 2, such as smoking, chewing tobacco, alcohol use, ADL, IADL, body mass index, physical activity, high cholesterol, hypertension, diabetes, and chronic heart disease. The current study shows that the prevalence of angina pectoris, COPD and asthma was 6.0%, 2.3% and 4.7%, respectively, among individuals aged 45 years and above in India. The prevalence of angina pectoris was higher among individuals with COPD (9.6% vs. 5.8%) and asthma (9.9% vs. 5.7%) than those without COPD and asthma, respectively. Additionally, angina pectoris was more prevalent among females and rural respondents with COPD (10.8% and 11.0%) and asthma (10.3% and 10.3%) compared to males and urban respondents with COPD (8.0% and 5.7%) and asthma (8.9% and 7.9%). Moreover, in the adjusted model, individuals with COPD (AOR 1.43, 95% CI 1.03 1.98) and asthma (AOR 1.44, 95% CI 1.17 1.77) had nearly 1.5-fold higher odds of having angina pectoris than those without COPD and asthma. The PSM estimates showed that individuals with COPD had 8.4% and 5.0% increased risk of angina pectoris compared to those without COPD in model 1 and model 2, respectively. We observed that, after adjusting to lifestyle, health-related and morbidity factors in model 2, both average treatment effect on untreated (ATU) and average treatment effect (ATE) values decreased by nearly 3.5%. Additionally, the PSM estimates demonstrated that individuals with asthma had a 3.4% and 2.9% increased risk of angina pectoris compared to those without asthma in model 1 and model 2, respectively. The study suggests that COPD and asthma are significantly associated with angina pectoris, and individuals with COPD and asthma have a higher risk of developing angina pectoris. Additionally, angina pectoris was more prevalent among females, rural respondents and adults aged 45–54 with COPD compared to males, urban respondents and those aged 65 and above, respectively, with COPD. Moreover, the findings of our study underscore the targeted primary and secondary interventions and team-based care approach among individuals with COPD and asthma to reduce the risk of CVD events in future.

## Introduction

Angina pectoris (AP) is a common clinical manifestation of coronary artery disease (CAD)^1–3^, characterized by chest discomfort associated with myocardial ischemia^4,5^, although in some cases, AP may result from non-CAD-related conditions, such as anemia, hyperthyroidism, respiratory diseases, and valvular diseases^2,6^. Existing literature suggests that the Rose Angina Questionnaire (RAQ) can be employed as a screening tool for individuals at risk of coronary heart disease in large-scale epidemiological surveys^7^. Previous literature demonstrated that angina in individuals during middle age significantly increases the risk of adverse outcomes, including mortality, myocardial infarction, heart failure, and other cardiovascular events^8^. Moreover, the application of intensive secondary preventive strategies in patients suffering from angina can effectively mitigate the potential occurrence of future cardiovascular events^9–11^.

Comorbidities are commonly observed at the time of diagnosis for both chronic obstructive pulmonary disease (COPD) and asthma^12–14^. Several studies demonstrated that individuals with chronic obstructive pulmonary disease (COPD) had a significantly higher prevalence of cardiovascular diseases compared to those without COPD^15–19^. The prevalence of cardiac disease among COPD patients varies between 14 and 33%^20,21^. Similarly, a prior study reported that patients with asthma showed a higher prevalence of ischemic heart disease (IHD) and heart failure (HF) compared to controls^16^.

Moreover, COPD poses a significant public health challenge, especially among individuals aged 40 years and above^20,22^. According to the Global Initiative for Chronic Obstructive Lung Disease (GOLD), the prevalence of COPD is expected to rise in low- and middle-income countries (LMICs) and the ageing populations in high-income countries^23^. A previous study suggests that the heightened risks of cardiovascular disease are especially notable among middle-aged to late-middle-aged individuals with COPD^17^.

A prior study demonstrated a significant link between asthma and an elevated risk of coronary vasospastic angina onset (CvsA)^24^. Similarly, previous studies and trials showed an association between asthma and coronary heart disease^16,25,26^. Moreover, the association between asthma and coronary heart disease was higher in females compared to males^25–27^. Further, previous investigations have revealed that systemic inflammation contributes to the elevated incidence of cardiovascular complications, particularly atherothrombosis, in patients with asthma^28,29^. Moreover, a prior study reported that ischemic heart disease (IHD) was associated with all-cause mortality among individuals with asthma^16^.

There is considerable evidence supporting the associations between COPD and cardiovascular diseases^16,18,30–32^. The association between COPD and Ischemic Heart Disease (IHD) exerts adverse consequences on overall prognosis. The development of both COPD and IHD is attributed to several shared factors, including exposure to inhaled noxious particles, hypoxia, systemic inflammation, endothelial dysfunction, heightened platelet reactivity, and increased arterial stiffness^33,34,34,35,35^. Notably, cardiovascular-related mortality in a significant portion of these patients follows shortly after an acute exacerbation of COPD^33^. Moreover, in the Towards a Revolution in COPD Health (TORCH) trial, cardiovascular deaths were observed in 26% of patients^36^. In the Understanding Potential Long-term Impacts on Function with Tiotropium (UPLIFT) trial, cardiovascular deaths accounted for 11% of patients and sudden cardiac death for 4.4% of patients with COPD^37^. A prior study demonstrated that men presenting with Rose angina exhibited an elevated risk of cardiovascular mortality or hospitalization, myocardial infarction, and heart failure when compared to those without angina^8^.

The coexistence of chronic lung diseases (COPD and asthma) and cardiovascular diseases is relatively common. Moreover, findings from a study indicate a strong association between COPD and major CVD morbidities, whereas a modest association between the diagnosis of asthma and various cardiovascular morbidities such as angina and coronary disease, cardiac arrhythmia, heart failure, cerebrovascular disease, other heart disease, or pulmonary embolism^12^. Understanding the effect of COPD and asthma on angina pectoris is crucial due to angina's role as a symptom of underlying heart disease, signifying reduced blood flow to the heart muscles. This exploration unveils the intricate interplay between respiratory and cardiovascular health. As COPD and asthma prevalence rises globally, especially in low- and middle-income countries (LMICs) with limited healthcare resources, comprehending their broader implications becomes vital; there exists a dearth of literature concerning the relationship of COPD and asthma with angina pectoris (AP) in LMIC settings, especially in India^24^. The current study utilizing nationally representative data uniquely addresses these concerns, offering insights beyond conventional health facility data. It holds special relevance for India's ageing population, providing evidence-based information to guide targeted interventions and addressing healthcare challenges linked to the coexistence of chronic lung diseases and cardiovascular conditions in this demographic. Therefore, the current study aimed to assess the effect of COPD and asthma on angina pectoris among individuals aged 45 years and above. Given the diagnostic and therapeutic implications, it is imperative to consider the coexistence of chronic lung diseases and angina pectoris in research and clinical practice. Rose angina can be a valuable tool to identify vulnerable subpopulations suffering from chronic lung diseases at higher risk of major cardiovascular events.

## Methods

### Data

**Figure 1:**
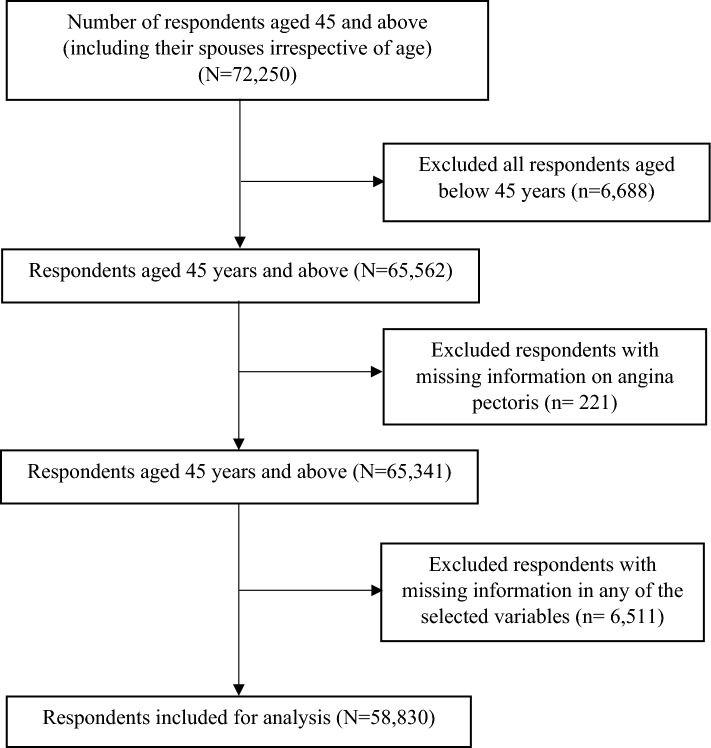
Study sample flowchart.

The data from the Longitudinal Ageing Study in India (LASI) Wave 1 (2017–2018) were used in this study. The survey collected data on the health, economic, and social factors, and consequences of India's population ageing. The LASI is a full-scale, nationally representative survey that included 72,250 individuals aged 45 years and older and their spouses (irrespective of age) across all states and union territories (UTs) of India except Sikkim. The LASI uses a multistage stratified area probability cluster sampling to select the eventual units of observation. This study presents scientific evidence on chronic health conditions, biomarkers, symptom-based health conditions, and functional and mental health. The LASI survey was conducted with a three-stage sampling design in rural areas and a four-stage sampling design in urban areas. In each state/UT, in the first stage, Primary Sampling Units (PSUs) (sub-district or Tehsils/Talukas) were selected, and in the second stage, villages in rural areas and wards in urban areas were selected in the selected PSUs. In the third stage, households were selected from each selected village; however, sampling in urban areas involved an additional stage, i.e., the random selection of one Census Enumeration Block (CEB) in each urban area. In the fourth stage, households were selected from each CEB. The main goal was to select a representative sample at each stage of sample selection. The detailed methodology and extensive information on the survey's design and data collection are available in the report^38^. The study encompasses 65,562 participants aged 45 years and above, excluding those below 45 years (n = 6688). After eliminating cases with missing information on angina pectoris (n = 221) and incomplete data in selected variables (n = 6511) (including hypertension: 15, diabetes: 18, physical inactivity: 54, ADL: 73, IADL: 132, body mass index: 6276 and any selected variables), the final sample size for analysis comprised 58,830 respondents (Fig. 1 presents the inclusion and exclusion criteria for the study sample).

### Measures

#### Outcome variable

The main outcome variable was symptom-based angina pectoris. The World Health Organization’s Rose Angina Questionnaire (Rose, 1962) was used for the symptom based assessment of angina pectoris. The Rose questionnaire is a screening tool for quantifying angina in population survey. Algorithm for the angina consists of 7 questions. Those who reported positive response for all the 7 questions or symptom are considered as having symptoms of angina pectoris.

### Treatment variable

The main explanatory variable was self-reported chronic lung diseases. In the LASI, respondents were asked, "Has any health professional ever told you that you have chronic lung diseases such as asthma, chronic obstructive pulmonary disease/chronic bronchitis or other chronic lung problems?". Additionally, for the analysis, we combined COPD and asthma as chronic lung diseases. In this study, we initially assessed the individual effects of asthma and chronic obstructive pulmonary disease (COPD). Subsequently, we examined the combined presence of at least one of these diseases.

### Covariates

We included eleven covariates in model 1 and further included eleven lifestyle and health-related covariates in model 2. Age was coded as 45–54 years, 55–64 years, 65–74 years and 75 + years. Sex was coded as male and female. Education was recoded as no education, primary, secondary, and higher. Marital status was coded as currently married, widowed, and others, not in a union. Working status was coded as never worked, currently working, and currently not working. Alcohol use was coded as ‘no’ and ‘yes’; smoking and chewing tobacco were coded as ‘never’, ‘former, and ‘current’. The body mass index (BMI) was computed by dividing the weight (in kilograms) by the square of the height (in meters). BMI was coded according to the criteria of the World Health Organisation’s classification; as underweight (< 18.5 kg/m^2^), normal weight (18.5–24.9 kg/m^2^), overweight (25.0–29.9 kg/m^2^), and obesity (≥ 30.0 kg/m^2^), for the analysis, overweight and obesity were combined^40^. The monthly per capita expenditure quintile (MPCE) or consumption quintile was categorized into five quintiles, poorest, poor, middle, rich, and richest (LASI collected information from households about their spending on food [a reference period of 7 days] and non-food items (reference periods of 30 and 365 days). After standardising the food and non-food expenditure to a 30-day reference period, the monthly per capita consumption expenditure [MPCE] was computed and used as the summary measure of consumption: poorest, poorer, middle, richer, and richest). Religion was categorized as Hindu, Muslim, Christian, and Others. The social group (caste) was categorized as Scheduled Castes (SC), Scheduled Tribes (ST), Other Backward Classes (OBC), and others. The ‘other’ category in caste is identified as having non SC/ST and OBC. The place of residence was coded as urban and rural. The regions were categorized as North, Central, East, Northeast, West, and South. In the LASI, respondents were asked, ‘Overall, how is your health in general? Would you say it is very good, good, fair, poor, or very poor?’ The responses were categorised into three groups as follows: good (‘very good’ and ‘good’), fair (moderate), and poor (‘poor’ and ‘very poor’). Activities of Daily Living (ADL) and Instrumental Activities of Daily Living (IADL) were coded as no and yes^41^. In LASI, respondents were asked, "Has any health professional ever diagnosed you with chronic heart diseases such as coronary heart disease (heart attack or Myocardial Infarction), congestive heart failure, or other chronic heart problems?”. The responses were coded as no and yes for chronic heart disease. Physical activity was categorized as physically active and physically inactive.

Weekly durations of both moderate and vigorous physical activities were computed: Moderate physical activity was defined as those who engaged in a minimum of 150 min of moderate-intensity physical activity in a week, while vigorous physical activity encompassed those who engaged in a minimum of 75 min of vigorous-intensity physical activities in a week. Respondents were subsequently categorized into two groups based on their engagement in moderate and vigorous activities: "Physically active," denoting those who engaged more than once a week, and "Physically inactive," characterizing individuals who engaged once a week or less often. Subsequently, a binary variable of physical activity variable was created as "Physically active," comprising those engaged in either moderate or vigorous physical activities, and "Physically inactive," encompassing those who were not engaged in any form of moderate or vigorous physical activity throughout the week^42,43^.

### Statistical analysis

Descriptive statistics and bivariate analysis were performed to examine the prevalence of angina among individuals with chronic lung diseases, COPD, and asthma with selected variables. Additionally, multivariable binary logistic regression analysis has been used to establish the association of COPD and asthma with angina pectoris. The survey weights were applied, and the findings were presented as adjusted odds ratio (AOR) and unadjusted odds ratio.

Moreover, propensity score matching (PSM) was employed to evaluate the effect of COPD, asthma, and chronic lung diseases on angina pectoris. Individuals with self-reported COPD, asthma and chronic lung diseases (either presence of COPD or asthma) were allocated into the treatment group and matched with counterparts in the control group through a nearest neighbour one matching technique with common support. This represents the counterfactual model used for the estimation of the effect of chronic lung diseases on angina pectoris by controlling the background characteristics selected for control as well as addressing potential biases due to the non-random allocation of subjects to either treatment or control group. The propensity score functions as a balancing score of the observed predictors, ensuring the similarity in the distribution of covariates between the treatment and comparison groups^44^. The computed propensity scores were based on individual and other background characteristics in two different models, Model 1 is matched for age, sex, education, marital status, working status, place of residence, caste, MPCE quintiles, religion, region, and self-rated health and Model 2 matched for additional variables namely smoking, chewing tobacco, alcohol use, ADL, IADL, body mass index, physical activity, high cholesterol, hypertension, diabetes, and chronic heart disease for COPD and asthma.

Furthermore, the Average Treatment Effect on the Treated (ATT) was calculated, which represents the average increase in the prevalence of angina pectoris among individuals with COPD, asthma and chronic lung diseases compared to their counterparts with similar background characteristics through matching. PSM analysis also calculated the Average Treatment Effect on the Untreated (ATU) and Average Treatment Effect (ATE).

A comparison of the average outcome for those with COPD/asthma/chronic lung diseases and those without is established using the propensity score matching (PSM) method. It compares the outcome (angina pectoris) between the treated and controlled observations given various background characteristics^45^.

$$p\left(X\right)={\text{Pr}}(D=1|X)$$

where $X$ is a multidimensional vector of pre-treatment characteristics, and $D=\{\mathrm{0,1}\}$ is the indicator variable of exposure to COPD/asthma/chronic lung diseases. The effect of COPD/asthma/chronic lung diseases for an individual $i$, denoted by ${\delta }_{i}$ is the difference between the potential outcome in the presence of COPD/asthma/chronic lung diseases (${Y}_{1}$) and the potential outcome in the absence of COPD/asthma/chronic lung diseases (${Y}_{0}$).

$${\delta }_{i}= {Y}_{1}-{Y}_{0}$$

where the average effect of COPD/asthma/chronic lung diseases on the outcome is provided by the Average Treatment Effect (ATE). This is given by

$$ATE=E\left({\delta }_{i}\right)=E({Y}_{1}-{Y}_{0})$$

A counterfactual model has been constructed to calculate the average effect of COPD/asthma/chronic lung diseases on the treated individuals. Using this model, the Average Treatment Effect on the treated (ATT) has been measured. It is the average increase in the outcome of those who had COPD/asthma/chronic lung diseases compared to those who weren’t having COPD/asthma/chronic lung diseases and had similar characteristics after matching.

$$ATT=E\left({Y}_{1}|D=1\right)-E({Y}_{0}|D=1)$$

where $E\left({Y}_{1}|D=1\right)$ is the average outcome of the treated individuals, and $E({Y}_{0}|D=1)$ is the average outcome the treated individuals would have obtained in the absence of treatment. Average treatment effect on the Untreated (ATU) calculated the average increase in the outcome of those who were not having COPD/asthma/chronic lung diseases as compared to those who had COPD/asthma/chronic lung diseases and had similar characteristics after matching.

$$ATU=E\left({Y}_{1}|D=0\right)-E({Y}_{0}|D=0)$$

where, $E\left({Y}_{1}|D=0\right)$ is the average outcome of the untreated individuals, and $E({Y}_{0}|D=0)$ is the average outcome the untreated individuals would have obtained in the presence of treatment.

To conduct the propensity score matching (PSM) analysis, the "*psmatch2*" command was employed using Stata (version 14.1). Additionally, the PS test was performed to evaluate the overall significance of the models after conducting a matching analysis in which pseudo-*R*2 shows an estimation of how well the predictors explain the probability of the outcome, and the p-value of the likelihood ratio test becomes insignificant or not in the matched sample. Additionally, we generated valid standard errors to produce valid treatment estimates with the bootstrap methods with 100, 500, 1000 replications. The result of Bootstrap confirms that there is a significant positive effect in all the reported models. All the analyses were conducted using Stata version 14.1.

### Ethics approval and consent to participate

The study was approved by the Indian Council of Medical Research (ICMR) Ethics Committee in January 2017 and written and oral informed consent was obtained from the participants. All methods were carried out in accordance with relevant guidelines and regulations and in accordance with the World Medical Association Declaration of Helsinki.

## Result

Table 1 presents the sample characteristics. A proportion of 34.57% of the participants were 65 years and older in this study. About 54.21% of the sample population was female. A large proportion of the sample (50.71%) had no education, and 73.84% were in marital union during the survey. Further, most of the sample population belonged to rural areas (69.92%). A proportion of 34.18% and 27.07% of the sample population were physically inactive and overweight/obese. Moreover, 27%, 11.92%, 3.83% and 5.85% of the sample reported to have hypertension, diabetes, COPD and asthma, respectively.

**Table 1:** Sample characteristics of the study population, LASI, 2017–2018.

Variables	N	%
Age groups		
45–54	21,826	34.97
55–64	18,269	30.41
65–74	13,061	24.07
75 +	5674	10.55
Gender		
Male	27,225	45.79
Female	31,605	54.21
Education level		
No education	27,645	50.71
Primary	14,685	23.32
Secondary	10,872	16.19
Higher	5628	9.77
Working status		
Never worked	16,103	26.13
Currently working	27,404	47.13
Currently not working	15,323	26.74
Marital status		
Currently married	44,068	73.84
Widowed	12,886	23.35
D/S/D/Others	1876	2.81
Place of residence		
Rural	38,522	69.92
Urban	20,308	30.08
Caste		
Scheduled caste	9910	19.44
Scheduled tribe	10,307	8.62
OBC	22,209	45.57
Others	16,404	26.37
MPCE quintile		
Poorest	11,596	21.09
Poorer	11,889	21.28
Middle	11,892	20.39
Richer	11,869	19.67
Richest	11,584	17.57
Region		
North	10,824	12.65
Central	8082	21.00
East	10,642	23.84
Northeast	7649	3.42
West	7702	15.83
South	13,931	23.26
Religion		
Hindu	43,170	82.55
Muslim	6979	11.02
Christian	5900	2.94
Others	2781	3.49
Self-rated health		
Good	24,309	37.87
Fair	24,659	44.29
Poor	9862	17.84
Difficulty in ADL		
No	50,871	84.48
Yes	7959	15.52
Difficulty in IADL		
No	39,736	63.49
Yes	19,094	36.51
Smoking tobacco		
Never	47,997	82.70
Former	2677	3.76
Current	8156	13.54
Chewing tobacco		
Never	45,906	76.48
Former	1445	2.38
Current	11,479	21.14
Alcohol use		
No﻿	48,230	84.85
Yes	10,600	15.15
BMI categories		
Normal	30,753	51.53
Underweight	10,912	21.40
Overweight/Obese	17,165	27.07
Physical inactivity		
Active	38,001	65.82
Inactive	20,829	34.18
Hypertension		
No	42,040	73.00
Yes	16,790	27.00
Diabetes		
No	51,334	88.08
Yes	7496	11.92
High cholesterol		
No	56,751	97.70
Yes	2079	2.30
Chronic heart diseases		
No	56,694	96.17
Yes	2136	3.83
COPD		
No	57,670	97.14
Yes	1160	2.86
Asthma		
No	56,535	94.15
Yes	2295	5.85
Lung diseases		
No	55,545	91.8
Yes	3285	8.2
Total	58,830	100.00

%: weighted column percentages to account for survey design and to provide national population estimates; ADL: Activities of daily living; IADL: Instrumental activities of daily living; MPCE: Monthly per capita consumption expenditure; COPD, chronic obstructive pulmonary diseases; BMI, body mass index.

Table 2 shows the prevalence of angina, COPD, asthma and chronic lung diseases among individuals aged 45 and above in India.

**Table 2:** Prevalence of angina pectoris and chronic lung diseases according to background characteristics.

Variables	Angina		COPD		Asthma		Lung diseases	
	N	Percentage (95% CI)	N	Percentage (95% CI)	N	Percentage (95% CI)	N	Percentage (95% CI)
Age groups								
45–54	1200	5.4 (5.0,5.9)	292	1.4 (1.2,1.6)	533	2.6 (2.3,3.0)	792	3.9 (3.5,4.3)
55–64	1076	6.3 (5.7,6.9)	360	2.2 (1.8,2.7)	634	5.1 (3.8,6.9)	947	6.8 (5.4,8.5)
65–74	823	6.3 (5.6,7.1)	326	3.2 (1.8,5.6)	715	5.8 (5.2,6.4)	994	8.5 (6.9,10.6)
75 +	370	6.0 (5.1,7.1)	182	3.2 (2.6,4.0)	413	7.8 (6.8,8.9)	552	10.0 (8.9,11.3)
Gender								
Male	1251	4.7 (4.3,5.1)	584	2.3 (2.0,2.5)	1155	5.3 (4.4,6.4)	1659	7.2 (6.2,8.3)
Female	2218	7.0 (6.5,7.5)	576	2.3 (1.6,3.3)	1140	4.2 (3.8,4.6)	1626	6.0 (5.2,7.0)
Education level								
No education	1888	6.9 (6.4,7.3)	518	2.0 (1.7,2.3)	1174	4.9 (4.5,5.4)	1598	6.4 (5.9,6.9)
Primary	925	6.4 (5.7,7.0)	341	2.4 (2.0,2.9)	664	5.2 (4.7,5.7)	948	7.1 (6.4,7.8)
Secondary	473	4.3 (3.7,5.0)	210	3.4 (1.5,7.4)	340	3.6 (2.8,4.6)	537	6.9 (4.6,10.1)
Higher	183	3.1 (2.4,3.9)	91	1.3 (0.9,1.7)	117	4.3 (1.6,11.6)	202	5.4 (2.4,11.9)
Working status								
Never Worked	1046	6.6 (5.9,7.3)	334	2.9 (1.6,5.1)	556	4.1 (3.5,4.8)	852	6.6 (5.0,8.6)
Currently working	1451	5.5 (5.1,6.0)	379	1.6 (1.3,1.9)	774	3.7 (2.9,4.8)	1095	4.9 (4.1,6.0)
Currently Not working	972	6.1 (5.6,6.7)	447	2.9 (2.5,3.3)	965	7.1 (6.5,7.7)	1338	9.3 (8.6,10.0)
Marital status								
Currently married	2534	5.9 (5.6,6.3)	814	2.0 (1.8,2.2)	1602	4.5 (3.9,5.3)	2308	6.1 (5.5,6.8)
Widowed	855	6.2 (5.6,6.9)	306	3.2 (1.8,5.7)	635	5.4 (4.7,6.1)	887	8.1 (6.4,10.2)
D/S/D/Others	80	4.1 (2.9,5.8)	40	1.4 (0.8,2.4)	58	3.5 (2.3,5.2)	90	4.5 (3.2,6.4)
Place of residence								
Rural	2570	6.7 (6.3,7.1)	757	2.1 (1.9,2.3)	1540	4.6 (4.3,4.9)	2184	6.3 (5.9,6.7)
Urban	899	4.2 (3.8,4.7)	403	2.7 (1.5,4.7)	755	5.0 (3.6,6.8)	1101	7.1 (5.3,9.5)
Caste								
Scheduled caste	592	6.1 (5.4,6.9)	212	2.0 (1.7,2.4)	426	4.8 (4.2,5.4)	610	6.4 (5.7,7.2)
Scheduled tribe	589	5.9 (5.2,6.8)	121	1.3 (0.9,1.9)	268	3.3 (2.7,4.0)	371	4.2 (3.5,5.1)
OBC	1296	5.7 (5.3,6.3)	487	2.6 (1.8,3.8)	978	5.2 (4.2,6.3)	1387	7.3 (6.0,8.8)
Others	992	6.2 (5.7,6.8)	340	2.2 (1.9,2.6)	623	4.3 (3.8,4.8)	917	6.1 (5.5,6.7)
MPCE quintile								
Poorest	630	5.8 (5.1,6.5)	170	1.3 (1.1,1.6)	406	4.2 (3.6,5.0)	555	5.3 (4.6,6.1)
Poorer	673	5.7 (5.1,6.4)	228	1.9 (1.6,2.3)	467	4.6 (4.0,5.2)	653	5.9 (5.3,6.7)
Middle	695	6.1 (5.4,7.0)	222	2.1 (1.7,2.5)	474	4.2 (3.7,4.8)	666	6.0 (5.3,6.7)
Richer	762	6.3 (5.6,7.1)	258	2.6 (2.0,3.3)	463	4.6 (3.8,5.4)	686	6.5 (5.7,7.4)
Richest	709	5.8 (5.2,6.6)	282	3.7 (1.9,7.2)	485	6.2 (4.1,9.2)	725	9.4 (6.6,13.3)
Region								
North	753	6.2 (5.6,6.9)	283	2.4 (2.1,2.8)	419	5.7 (5.0,6.5)	687	7.9 (7.1,8.8)
Central	583	7.2 (6.4,8.0)	151	2.1 (1.7,2.6)	292	3.8 (3.2,4.5)	423	5.7 (4.9,6.5)
East	567	5.8 (5.1,6.6)	165	1.8 (1.5,2.2)	475	4.6 (4.1,5.1)	597	5.9 (5.4,6.5)
Northeast	452	6.0 (5.3,6.8)	69	0.8 (0.6,1.1)	163	2.4 (1.9,3.0)	223	3.1 (2.5,3.7)
West	528	8.1 (7.2,9.1)	92	1.4 (1.1,1.8)	350	5.0 (4.3,5.8)	418	6.0 (5.2,6.8)
South	586	3.4 (3.0,3.9)	400	3.6 (2.1,6.1)	596	5.2 (3.5,7.6)	937	8.1 (5.8,11.2)
Religion								
Hindu	2540	5.8 (5.4,6.1)	831	2.2 (1.7,2.8)	1783	4.7 (4.2,5.4)	2472	6.5 (5.7,7.3)
Muslim	455	7.4 (6.4,8.6)	174	2.6 (2.0,3.3)	315	4.9 (4.2,5.7)	470	7.0 (6.1,8.1)
Christian	323	4.7 (3.6,6.1)	82	1.9 (1.2,3.0)	133	4.1 (2.8,5.9)	209	5.9 (4.3,8.0)
Others	151	7.0 (5.5,8.8)	73	3.4 (2.4,4.9)	64	3.6 (2.5,5.3)	134	6.5 (5.0,8.5)
Self-rated health								
Good	865	3.7 (3.3,4.1)	230	1.0 (0.8,1.3)	473	2.4 (2.0,2.8)	677	3.3 (2.9,3.8)
Fair	1632	6.5 (6.0,7.0)	501	2.5 (1.6,3.7)	948	4.7 (3.7,5.8)	1380	6.7 (5.4,8.2)
Poor	972	9.4 (8.5,10.3)	429	4.4 (3.8,5.0)	874	9.8 (8.9,10.7)	1228	13.1 (12.1,14.1)
Difficulty in ADL								
No	2747	5.4 (5.1,5.7)	927	2.1 (1.6,2.8)	1785	4.3 (3.8,5.0)	2595	6.1 (5.4,7.0)
Yes	722	9.1 (8.1,10.2)	233	3.0 (2.5,3.7)	510	6.6 (5.9,7.5)	690	8.7 (7.8,9.7)
Difficulty in IADL								
No	1970	5.1 (4.8,5.5)	635	1.6 (1.4,1.9)	1197	3.8 (3.1,4.6)	1766	5.1 (4.4,5.9)
Yes	1499	7.4 (6.8,8.1)	525	3.4 (2.4,4.9)	1098	6.3 (5.7,6.9)	1519	9.0 (7.8,10.4)
Smoking tobacco								
Never	2818	6.0 (5.6,6.4)	844	2.1 (1.6,2.7)	1710	4.4 (3.8,5.0)	2430	6.0 (5.3,6.9)
Former	161	6.2 (4.9,7.9)	113	4.1 (3.2,5.3)	222	10.7 (8.9,12.9)	317	13.7 (11.7,16.0)
Current	490	5.7 (5.0,6.5)	203	2.9 (2.3,3.6)	363	5.0 (4.4,5.7)	538	7.5 (6.7,8.4)
Chewing tobacco								
Never	2703	5.9 (5.5,6.3)	933	2.4 (1.8,3.0)	1761	4.8 (4.2,5.5)	2565	6.7 (5.9,7.7)
Former	90	7.0 (4.7,10.5)	48	3.2 (2.1,4.8)	87	5.9 (4.4,7.7)	125	8.2 (6.4,10.4)
Current	676	6.1 (5.5,6.8)	179	1.8 (1.5,2.3)	447	4.1 (3.6,4.6)	595	5.6 (5.0,6.3)
Alcohol Use								
No	2941	6.2 (5.8,6.6)	951	2.2 (1.7,2.9)	1834	4.6 (4.1,5.3)	2650	6.5 (5.7,7.3)
Yes	528	4.7 (4.2,5.3)	209	2.4 (1.9,3.0)	461	5.0 (4.4,5.7)	635	7.0 (6.2,7.8)
BMI categories								
Normal	1738	5.9 (5.4,6.4)	544	1.7 (1.5,1.9)	1065	4.4 (3.6,5.4)	1531	5.8 (4.9,6.7)
Underweight	702	6.4 (5.8,7.1)	283	3 (2.5,3.5)	642	6.5 (5.8,7.2)	872	8.8 (8.0,9.7)
Overweight/obese	1029	5.7 (5.2,6.3)	333	2.8 (1.5,5.0)	588	3.9 (3.3,4.7)	882	6.2 (4.7,8.1)
Physical inactivity								
Active	2269	5.9 (5.5,6.3)	643	2.1 (1.5,3.0)	1291	4.2 (3.5,5.0)	1853	6.0 (5.1,7.0)
Inactive	1200	6.1 (5.6,6.6)	517	2.5 (2.2,2.9)	1004	5.7 (5.2,6.2)	1432	7.6 (7.1,8.3)
Hypertension								
No	2107	5.3 (4.9,5.6)	678	1.7 (1.5,1.9)	1428	4.2 (3.6,4.9)	2008	5.6 (4.9,6.3)
Yes	1362	7.8 (7.1,8.5)	482	3.8 (2.4,5.9)	867	6.1 (5.4,6.8)	1277	9.1 (7.6,11.0)
Diabetes								
No	2963	5.9 (5.6,6.2)	974	2.0 (1.8,2.2)	1945	4.6 (4.1,5.2)	2769	6.2 (5.7,6.8)
Yes	506	6.4 (5.5,7.4)	186	4.3 (1.8,9.9)	350	5.3 (4.3,6.7)	516	8.9 (5.9,13.2)
High Cholesterol								
No	3286	5.9 (5.6,6.2)	1067	2.2 (1.8,2.8)	2179	4.7 (4.2,5.2)	3082	6.5 (5.8,7.2)
Yes	183	8.5 (6.8,10.6)	93	3.6 (2.5,5.1)	116	6.0 (4.3,8.2)	203	9.3 (7.3,11.8)
Chronic heart diseases								
No	3156	5.6 (5.3,5.9)	1048	2.0 (1.8,2.2)	2132	4.6 (4.1,5.1)	3033	6.1 (5.6,6.7)
Yes	313	14.9 (12.0,18.5)	112	9.9 (3.3,26.3)	163	8.2 (6.3,10.7)	252	16.8 (9.1,29.1)
COPD								
No	3333	5.9 (5.6,6.2)						
Yes	136	9.5 (7.0,12.9)						
Asthma								
No	3234	5.8 (5.5,6.1)						
Yes	235	9.5 (7.8,11.6)						
Lung diseases								
No	3118	5.7 (5.4,6.0)						
Yes	351	9.6 (8.0,11.3)						
Total	3469	6.0 (5.6,6.3)	1160	2.3 (1.8,2.8)	2295	4.7 (4.2,5.2)	3285	6.5 (5.9,7.2)

weighted row %: weighted row percentages to account for survey design and to provide national population estimates; COPD, chronic obstructive pulmonary diseases; BMI, body mass index; MPCE, monthly per capita expenditure, ADL, activities of daily living; IADL, instrumental activities of daily living.

### The prevalence of angina pectoris

The prevalence of angina pectoris was 6.0% (95% Confidence Interval (CI) 5.6, 6.3). Our results show that the prevalence of angina was higher among female respondents (7.0% vs. 4.7%) and individuals aged 55–74 years (6.3% vs. 5.4%) compared to male respondents and those aged 45–54, respectively. Additionally, we found that angina was more prevalent among individuals with no education (6.9% vs. 3.1%) than those with higher education. Notably, the results show that the prevalence of angina was higher among individuals from rural areas (6.7% vs. 4.2%) than those from urban areas.

Moreover, our findings show that the prevalence of symptom-based angina pectoris was higher among individuals with COPD (9.6% vs. 5.8%) and asthma (9.9% vs. 5.7%) than those without COPD and asthma, respectively. Moreover, the angina pectoris was more prevalent among individuals with chronic heart diseases (14.9% vs. 5.6%), hypertension (7.8% vs. 5.3), diabetes (6.4% vs. 5.9%), and higher cholesterol (8.5% vs. 5.9%) than those without chronic heart diseases, hypertension, diabetes and higher cholesterol, respectively. Additionally, we found that the prevalence of angina was higher among individuals with difficulty in ADL (9.1% vs. 5.4%), and IADL (7.4% vs. 5.1%) than those without difficulty in ADL and IADL, respectively.

### The prevalence of COPD, asthma and chronic lung diseases

Our findings illustrate that the prevalence of COPD, asthma and chronic lung diseases was 2.3% (1.8, 2.8), 4.7% (4.2, 5.2) and 6.5% (5.9, 7.2). We observed that the prevalence of COPD and asthma increased with age, and COPD was to be higher among individuals aged 65 years and above (3.2% vs. 1.4%), and asthma was more prevalent among individuals aged 75 years and above (7.8% vs. 2.6%) than those aged 45–54 years. There was no difference in the prevalence of COPD among male and female respondents, while asthma was more prevalent among male (5.4% vs. 4.2%) than female respondents. However, the overall prevalence of chronic lung diseases was higher among male (7.2% vs. 6.0%) than female respondents. The prevalence of COPD and asthma was 2.7% and 5.0%, respectively, among individuals from urban areas.

Additionally, our result shows that the prevalence of COPD and asthma was higher among former smokers (4.1% and 10.7%) than never smokers (2.1% and 4.4%). We observed that COPD and asthma were more prevalent among individuals with difficulty in ADL (3.0% and 6.6%) and IADL (3.4% and 6.3%) than those without difficulty in ADL (2.1% and 4.3%) and IADL (1.6% and 3.8%), respectively.

Moreover, the prevalence of COPD and asthma was higher among individuals with hypertension (3.8% and 6.1%), diabetes (4.3% and 5.3%), chronic heart diseases (9.9% and 8.2%), and higher cholesterol (3.6% and 6.0%) than individual without hypertension (1.7% and 4.2%), diabetes (2.0% and 4.2%), chronic heart diseases (2.0% and 4.6%) and high cholesterol (2.2% and 4.7%), respectively. In addition, we observed that the overall prevalence of chronic lung diseases was significantly higher among individuals with chronic heart diseases (16.8% vs. 6.1%) than those without chronic heart diseases.

### The prevalence of angina pectoris among individuals with COPD and asthma by gender, place of residence and age categories

**Figure 2:**
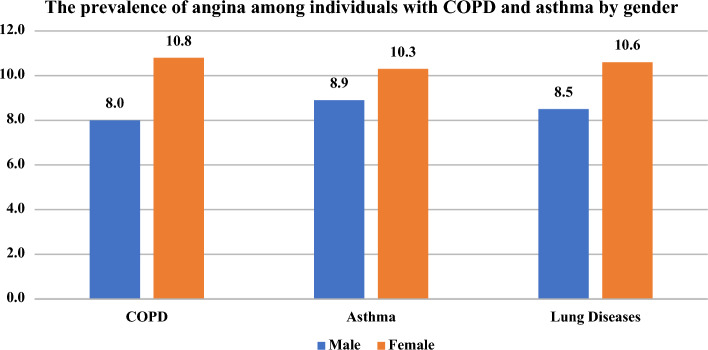
The prevalence of Angina pectoris among individuals with COPD and asthma by gender.

Figure 2 shows that the angina pectoris was more prevalent among female respondents with COPD (10.8% vs. 8.0%) and asthma (10.3% vs. 8.9%) than male respondents.

**Figure 3:**
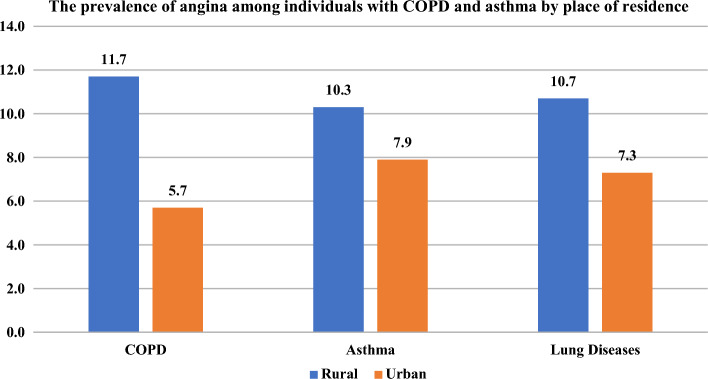
The prevalence of angina pectoris among individuals with COPD and asthma by place of residence.

Figure 3 demonstrates that the prevalence of angina pectoris was significantly higher among rural respondents with COPD (11.7% vs 5.7%) and asthma (10.3% vs. 7.9%) than those from urban areas.

**Figure 4:**
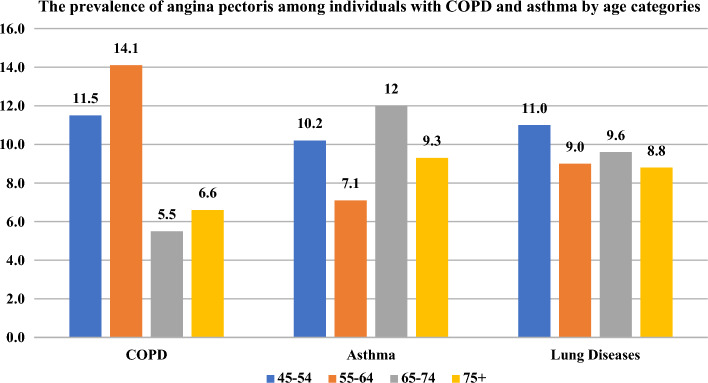
The prevalence of angina pectoris among individuals with COPD and asthma by age categories.

Figure 4 presents that the prevalence of angina pectoris was significantly higher among individuals aged 45–54 and 55–64 with COPD (11.5% vs.14.1%) than those aged 65–74 and 75 + (5.5% and 6.6%).

Table 3 Unadjusted and adjusted odds ratio of angina pectoris using multivariable logistic regression analysis is given in Table 3. This table displays the odds ratio and its 95% CI estimate with respective angina pectoris in COPD, Asthma, Lung Disease and various socio-economic, demographic and lifestyle variables categories in comparison to the reference category. The results show that in the adjusted models, individuals with COPD (AOR: 1.43, 95% CI 1.03 1.98) and asthma (AOR: 1.44, 95% CI 1.17 1.77) had higher odds of having angina pectoris than those without COPD and asthma, respectively. Moreover, in the adjusted models, findings show that individuals with hypertension (AOR: 1.32, 95% CI 1.15 1.51), and chronic heart diseases (AOR: 2.55, 95% CI 1.99 3.28) had higher odds of having angina pectoris than those without hypertension, cholesterol, and chronic heart diseases, respectively.

**Table 3:** Logistic regression estimates for angina pectoris among older adults in India, LASI 2017–2018.

Variables	Angina pectoris	
	Unadjusted odds ratio (95% CI)	Adjusted odds ratio (95% CI)
Lung diseases		
No	Ref	Ref
Yes	1.75*** (1.43 2.13)	1.48*** (1.22 1.78)
COPD		
No	Ref	Ref
Yes	1.69*** (1.20 2.38)	1.43* (1.03 1.98)
Asthma		
No	Ref	Ref
Yes	1.72*** (1.37 2.15)	1.44*** (1.17 1.77)
Hypertension		
No		Ref
Yes		1.32*** (1.15 1.51)
Diabetes		
No		Ref
Yes		1.00 (0.84 1.19)
High cholesterol		
No		Ref
Yes		1.32* (1.00 1.73)
Heart diseases		
No		Ref
Yes		2.55*** (1.99 3.28)

*p < 0.05, **p < 0.01, ***p < 0.001; CI, Confidence Interval; Ref, reference category; COPD, chronic obstructive pulmonary diseases.

adjusted odds ratio, adjusted for age, sex, education, marital status, working status, place of residence, caste, MPCE quintiles, religion, region, self-rated health, smoking, chewing tobacco, alcohol use, ADL, IADL, body mass index.

Table 4 represents the propensity score matching analysis results for the effect of COPD and asthma on angina pectoris among individuals aged 45 years and above in India. We presented the PSM estimates in two different models. Model 1 is adjusted for age, sex, education, marital status, working status, place of residence, caste, MPCE quintiles, religion, region, and self-rated health and Model 2 adjusted for additional variables, namely smoking, chewing tobacco, alcohol use, ADL, IADL, body mass index, physical activity, high cholesterol, hypertension, diabetes, and chronic heart disease.

**Table 4:** The matching estimates of the effect of COPD and asthma on angina pectoris.

Variable	Sample	Treated	Controls	Difference	S.E	T-stat	P-Value
Model 1							
COPD							
Angina	Unmatched	0.117	0.058	0.059	0.007	8.52	
	ATT	0.117	0.073	0.044	0.014*	3.55	0.002*
	ATU	0.058	0.142	0.085			
	ATE			0.084			
*based on 1000 reps bootstrap standard error (S.E. = ATT: 0.012; 100 reps: 0.013; 500 reps: 0.015)							
Asthma							
Angina	Unmatched	0.102	0.057	0.045	0.005	9.02	
	ATT	0.102	0.073	0.029	0.010*	3.37	0.003*
	ATU	0.057	0.092	0.034			
	ATE			0.034			
*based on 1000 reps bootstrap standard error (S.E. = ATT: 0.009; 100 reps: 0.010; 500 reps: 0.010)							
CLD							
Angina	Unmatched	0.107	0.056	0.051	0.004	12.00	
	ATT	0.107	0.063	0.044	0.008*	6.04	0.000*
	ATU	0.056	0.093	0.036			
	ATE			0.037			
*based on 1000 reps bootstrap standard error (S.E. = ATT: 0.007; 100 reps: 0.009; 500 reps: 0.008)							
Model 2							
COPD							
Angina	Unmatched	0.117	0.058	0.059	0.007	8.52	
	ATT	0.117	0.068	0.049	0.014*	4.05	0.001*
	ATU	0.058	0.108	0.050			
	ATE			0.050			
*based on 1000 reps bootstrap standard error (S.E. = ATT: 0.012; 100 reps: 0.016; 500 reps: 0.015)							
Asthma							
Angina	Unmatched	0.102	0.057	0.045	0.005	9.02	
	ATT	0.102	0.082	0.020	0.010*	2.35	0.000
	ATU	0.057	0.087	0.029			
	ATE			0.029			
*based on 1000 reps bootstrap standard error (S.E. = ATT: 0.009; 100 reps: 0.010; 500 reps: 0.010), p-value = 1000 reps, 0.047							
CLD							
Angina	Unmatched	0.107	0.056	0.051	0.004	12.00	
	ATT	0.107	0.069	0.037	0.009*	5.20	0.000*
	ATU	0.056	0.095	0.039			
	ATE			0.039			
*based on 1000 reps bootstrap standard error (S.E. = ATT: 0.007; 100 reps: 0.009; 500 reps: 0.008)							

*COPD,* chronic obstructive lung diseases; *CLD,* chronic lung diseases; ATT, average treatment effect on the treated; ATU, average treatment effect on the untreated, ATE, average treatment effect.

Model 1, covariates: age, sex, education, marital status, working status, place of residence, caste, MPCE quintiles, religion, region, and self-rated health.

Model 2, covariates: age, sex, education, marital status, working status, place of residence, caste, MPCE quintiles, religion, region, self-rated health, smoking, chewing tobacco, alcohol use, ADL, IADL, physical activity, body mass index, high cholesterol, hypertension, diabetes, and chronic heart diseases.

### Effect of COPD on angina pectoris.

The unmatched sample estimates for angina pectoris indicate that individuals with COPD had a 5.9% higher chance of having angina pectoris than those without COPD. Using the nearest neighbour matching with common support, the Average treatment effect on the treated (ATT) values illustrate that, on an average, angina pectoris among individuals with COPD were 4.4% and 4.9% higher in model 1 and model 2, respectively, than the matched control group. Additionally, the Average Treatment Effects on the Untreated (ATU) values demonstrate that among those who didn’t have COPD (control group), if they were suffering from COPD, their chances of having angina pectoris were likely to increase by 8.5% and 5.0% in model 1 and model 2, respectively.

Notably, the Average Treatment Effect (ATE) estimates were 0.084 and 0.050 in model 1 and model 2, respectively. The ATE value suggest that, on an average there is 8.4% and 5.0% higher chances of having angina pectoris among individual with COPD in model 1 and mode 2, respectively. We observed that, after adjusting to lifestyle, health-related and morbidity factors in model 2, both ATU and ATE values decreased by nearly 3.5%.

### Effect of asthma on angina pectoris

The unmatched sample estimates for angina pectoris indicate that individuals with asthma had a 4.5% higher chance of having angina pectoris than those without asthma. The ATT values illustrate that on an average, angina pectoris among individuals with asthma was 2.9% and 2.0% higher in model 1 and model 2, respectively, than the matched control group. Moreover, the ATU values show that among those who didn’t have asthma, if they were suffering from asthma, their chances of having angina pectoris were likely to increase by 3.4% and 2.9% in model 1 and model 2, respectively.

Furthermore, the ATE estimates were found to be 0.034 and 0.029 in model 1 and model 2, respectively. The ATE value suggests that, on an average there is 3.4% and 2.9% higher chances of having angina pectoris among individual with asthma in model 1 and mode 2, respectively. We observed that after adjusting to lifestyle, health-related and morbidity factors, ATU and ATE values decreased by 0.5% in model 2.

### Effect of chronic lung diseases on angina pectoris

The unmatched sample estimates for angina pectoris indicate that individuals with chronic lung diseases had a 5.1% higher chance of having angina pectoris than those without chronic lung diseases. The ATT values present that, on an average, angina pectoris among individuals with chronic lung diseases was 4.4% and 3.7% higher in model 1 and model 2, respectively, than the matched control group. The results show that the ATU values show that among those who didn’t have chronic lung diseases, if they were suffering from chronic lung diseases, their chances of having angina pectoris was likely to increase by 3.6% and 3.9% in model 1 and model 2, respectively. Moreover, The ATE value suggests that, on an average there is 3.7% and 3.9% higher chances of having angina pectoris among individual with asthma in model 1 and model 2, respectively.

Table 5 presents the overall significance of the model after conducting a matching analysis and illustrate the value of pseudo *R2*, likelihood ratio, *X2* and the *p*-value of the likelihood ratio test for unmatched and matched sample. The pseudo *R*2 depicts an estimation of how well the predictors explain the probability of the outcome. After matching, pseudo *R2* value decreases from 0.056 to 0.004 for COPD and 0.065 to 0.003 for asthma in model 1 and 0.067 to 0.008 for COPD and 0.071 to 0.004 for asthma in model 2. Moreover, the *p-*value of the likelihood (LR) ratio test in the matched sample becomes insignificant in model 1 (*p* > chi^2^ = 0.996 for COPD and *p* > chi^2^ = 0.888 for asthma) and model 2 (*p* > chi^2^ = 0.974 for COPD and *p* > chi^2^ = 0.994 for asthma).

**Table 5:** Overall significance of the model after conducting matching analysis.

Treatment	Sample	Pseudo R2	LR chi^2^	*p* > chi^2^
Model 1				
CLD	Unmatched	0.065	1654.55	0.000
	Matched	0.002	22.54	0.797
COPD	Unmatched	0.056	643.31	0.000
	Matched	0.004	12.74	0.996
Asthma	Unmatched	0.065	1,257	0.000
	Matched	0.003	20.15	0.888
Model 2				
CLD	Unmatched	0.073	1849.56	0.000
	Matched	0.003	31.03	0.913
COPD	Unmatched	0.067	763.43	0.000
	Matched	0.008	26.92	0.974
Asthma	Unmatched	0.071	1,370	0.000
	Matched	0.004	23.28	0.994

*if B > 25%, R outside [0.5; 2].

## Discussion

The current study assessed the effect of COPD and asthma on angina pectoris using the propensity score matching method by constructing a control group with two different models. The study shows that COPD and asthma are significantly associated with angina pectoris, and individuals with COPD and asthma have higher chances of having angina pectoris even after adjustment with potential confounding variables such as lifestyle, health-related and morbidity factors. Our results illustrate that individuals with COPD and asthma have a higher prevalence of angina pectoris than those without COPD and asthma. Multiple studies demonstrated that patients with COPD and asthma had a higher prevalence of cardiovascular diseases compared to controls^16,21,31,46^. Our study revealed a higher prevalence of angina pectoris in females with COPD compared to males with COPD, suggesting an increased risk of heart disease in female respondents. This finding aligns with recent research demonstrating a greater prevalence of angina pectoris among females than males^47^. One prior study reported that the odds ratio for angina and coronary disease was higher among women diagnosed with asthma compared to men^12^. A previous study reported that the incidence of angina rises concomitantly with advancing age, a pattern observed in both male and female populations. It is worth noting that women tend to develop coronary artery disease at a later stage of life in comparison to their male counterparts^48^.

We observed that the prevalence of angina pectoris was significantly higher among individuals aged 45–54 and 55–64 years with COPD than those aged 65–74 and 75 years and above. Our results indicated that the risk of cardiovascular events may be higher among younger adults suffering from COPD. One prior study reported that among patients diagnosed with COPD, the odds ratio for angina and coronary diseases, heart failure, and cerebrovascular diseases was higher among the age group 35–44 years, and a significant reduction was observed in the older age cluster^12^. Similarly, we observed that the prevalence of COPD and asthma was higher among individuals from urban areas. On the other hand, the prevalence of angina pectoris was significantly higher among rural respondents with COPD and asthma than those from urban areas. The current study findings further corroborated that individuals with COPD and asthma living in rural areas may be at higher risk of future cardiovascular events. One prior study reported that socioeconomically disadvantaged patients were found to be more likely to have angina; however, they were less likely to visit general practitioners^3^.

Our findings suggest that COPD and asthma were significantly associated with angina pectoris. In the adjusted model, individuals with COPD and asthma had nearly 1.5 times higher odds of having angina pectoris. Moreover, in the adjusted model 2, PSM estimates demonstrate that individuals with COPD and asthma have a significantly 4.9% and 2.9% higher chance of having angina pectoris, respectively, compared to the matched control group. Consistently, a prior study demonstrated that patients with COPD had a 1.7-fold increased risk of angina compared to those without COPD^14^. One previous study showed that asthma was independently associated with coronary vasospastic angina (CVsA)^24^. In addition, prior reports have noted the concurrent presence of CVsA in individuals with asthma^24,49^.

Importantly, we observed that, after adjustment to various factors, ATE value decreased from 8.4% in model 1 to 5.0% in model 2, demonstrating the observed differences in the estimates due to adjustment with lifestyle and morbidity factors. COPD and coronary artery disease are highly prevalent chronic conditions; both conditions share common risk factors^33,50^. COPD is characterized by progressive airflow limitation due to chronic airway inflammation^50^. Airflow limitations (AL) are prevalent among individuals with cardiovascular disease (CVD), especially in those with hospital-based coronary artery diseases (CAD). However, AL is frequently underdiagnosed and consequently undertreated^51^. Prior evidence indicated that individuals with airflow limitations have a markedly elevated risk of mortality due to myocardial infarction^52^. Moreover, previous studies demonstrated that individuals with COPD had an increased risk of myocardial infarction, angina, coronary heart disease, and congestive heart failure^12,14,18,31,53,54^.

In the adjusted model 2, the ATE estimates for asthma decreased from 3.4 to 2.9%, indicating no major differences due to adjustment with the lifestyle and morbidity factors. In multiple previous studies, asthma was found to be associated with an increased risk of major CVD (myocardial infarction, angina, coronary heart disease, cerebrovascular disease, heart failure, and all-cause mortality)^12,55,56^. The presence of asthma in individuals could potentially elevate the risk of CVD and metabolic disorders, which can be attributed to the involvement of shared inflammatory mediators that play a role in developing CVD and insulin resistance^55^.

### Clinical implication

Our study has potential implications for practice and future research for the primary and secondary prevention of cardiovascular events among individuals with COPD and asthma. Chest pain has commonly been associated with ischemia, and existing literature suggests that in patients with coronary artery disease, a substantial proportion, approximately 70–80% of episodes of ischemia remain asymptomatic^57^. A recent study reported a case with multiple previous complications, including COPD, chronic kidney disease, MI, and coronary artery bypass graft surgery, with recent complaints on worsening angina^33^. Compared to patients diagnosed with IHD without concomitant COPD, individuals with COPD exhibit more complex coronary conditions and a notably worse prognosis^58^. The detrimental effect on prognosis is evident as the elevation of troponin levels associated with COPD exacerbation is an independent negative prognostic indicator, and it is more prevalent among patients with IHD^59^. Additionally, the pharmacological treatment of these patients is intricate and several studies have highlighted that COPD patients are usually undertreated due to concerns regarding potential adverse effects, particularly those related to β-blockers^34,58,60^. Interestingly, in this context, ivabradine could be highly beneficial for angina symptom management and quality life, given its efficacy in patients with both asthma and COPD, without inducing any adverse effects on respiratory function or symptoms. Moreover, it has been demonstrated to enhance exercise capacity and functional class in individuals diagnosed with COPD^33^.

### Strengths and limitations

Our study boasts several notable strengths. Firstly, the current study is the first population-based cross-sectional study with a large sample size that evaluated the effect of COPD and asthma on angina pectoris in LMIC settings, especially in India. Additionally, the data collection process was robust, with LASI incorporating a set of additional questions for participants reporting a medical professional diagnosis. This inclusive approach covered crucial details like the diagnosing physician, date of diagnosis, and current treatment status, enhancing the depth and validity of our findings. Despite these strengths, our study has certain limitations that warrant acknowledgment. The cross-sectional nature of the study precludes the establishment of a causal relationship, emphasizing the importance of interpreting findings cautiously. Another key limitation is the reliance on self-reported data for COPD and asthma, introducing the potential for reporting and recall bias. There is a possibility of recall bias that cannot be entirely eliminated. It is essential to recognize these limitations while interpreting the findings of this study. Additionally, further investigation should be employed using robust methodologies, including prospective cohort studies or randomized controlled trial to strengthen and validate the observed relationships.

## Conclusion

The findings suggest that COPD and asthma are significantly associated with elevated risk of angina pectoris, which may further lead to adverse cardiovascular events in future. Additionally, angina pectoris was found to be more prevalent among females, rural respondents and adults aged 45–59 with COPD compared to males, urban respondents and those aged 60 and above, respectively, with COPD. Identifying vulnerable subpopulations suffering from COPD and asthma at higher risk of future cardiovascular events using the rose angina questionnaire is imperative for tailored primary and secondary prevention approaches. The strategies may involve early diagnosis of respiratory diseases and the optimization of treatment approaches, ensuring that funds are appropriately allocated to mitigate the impact of chronic lung diseases on the risk of cardiovascular diseases and adverse health outcomes. A holistic approach to care will optimize patient outcomes and quality of life for individuals with COPD and asthma. Integrating preventive measures and comorbidity management strategies is essential for patients with specific risk factors like high cholesterol, hypertension, and diabetes to improve overall health and prevent CVD complications. Under uncertainty in the level of significance of the sample size or any other parameter, the current study can be extended using neutrosophic statistics as future research^61,62^

## Data Availability

The datasets used and/or analysed during the current study are available in the repository of the Gateway to Global Aging Data (https://g2aging.org/ ).
